# Hollow Au-Ag Nanoparticles Labeled Immunochromatography Strip for Highly Sensitive Detection of Clenbuterol

**DOI:** 10.1038/srep41419

**Published:** 2017-01-30

**Authors:** Jingyun Wang, Lei Zhang, Youju Huang, Anirban Dandapat, Liwei Dai, Ganggang Zhang, Xuefei Lu, Jiawei Zhang, Weihua Lai, Tao Chen

**Affiliations:** 1 State Key Laboratory of Food Science and Technology, Nanchang University, Nanchang 330047, China; 2 Division of Polymer and Composite Materials, Ningbo Institute of Material Technology and Engineering Chinese Academy of Sciences, No. 1219 Zhongguan West Road, Zhenhai District, Ningbo 315201, China; 3 Department of Biotechnology, Kumaun University, Bhimtal-263136, Uttarakhand, India

## Abstract

The probe materials play a significant role in improving the detection efficiency and sensitivity of lateral-flow immunochromatographic test strip (ICTS). Unlike conventional ICTS assay usually uses single-component, solid gold nanoparticles as labeled probes, in our present study, a bimetallic, hollow Au-Ag nanoparticles (NPs) labeled ICTS was successfully developed for the detection of clenbuterol (CLE). The hollow Au-Ag NPs with different Au/Ag mole ratio and tunable size were synthesized by varying the volume ratio of [HAuCl4]:[Ag NPs] via the galvanic replacement reaction. The surface of hollow Ag-Au NPs was functionalized with 11-mercaptoundecanoic acid (MUA) for further covalently bonded with anti-CLE monoclonal antibody. Overall size of the Au-Ag NPs, size of the holes within individual NPs and also Au/Ag mole ratio have been systematically optimized to amplify both the visual inspection signals and the quantitative data. The sensitivity of optimized hollow Au-Ag NPs probes has been achieved even as low as 2 ppb in a short time (within 15 min), which is superior over the detection performance of conventional test strip using Au NPs. The optimized hollow Au-Ag NPs labeled test strip can be used as an ideal candidate for the rapid screening of CLE in food samples.

Clenbuterol (CLE), a beta 2-adrenergic agonists, is one of the most popular drugs often used by individuals, especially athletes and bodybuilders who are looking to lose weight. CLE is indeed an effective thermogenic and anti-catabolic which helps to lose weight quickly. CLE is also commonly used for asthma treatment in humans^1^. However, it has several serious side effects including cardiac arrhythmia or even sudden death. CLE is also found to promote animal growth by increasing muscular mass and decreasing fat accumulation at the applied dosage (e.g., ten to hundred times higher than the normally therapeutic usage), which leads to an illegal use of CLE in meat industry for the economic interest^2^. Accumulative residues of CLE in animal tissues can result in clinical symptom such as cardiac palpitation, tachycardia, nervousness, muscle tremors and confusion^3^. Due to these serious health issues, the use of CLE as feed additive has been banned in many countries including China, United States and most European countries. This also in turn requires a great demand for a rapid, accurate sensor for testing CLE, which is an urgent and challenging task for food safety.

In the past few decades, numerous efforts were devoted to establish various methods for the detection of CLE residues in biological matrices, including liquid chromatographic mass spectrometry^4^,^5^,^6^, gas chromatographic mass spectrometry^7^,^8^, high performance liquid chromatographic^9^,^10^ and enzyme-linked immunosorbent assay^11^. Although these methods have their own advantages in high sensitivity and selectivity, most of the reports are time-consuming, and require tedious sample pretreatment, expensive equipment and skilled workers, which limit its practical use, especially in on-site detection.

From the standpoint of on-site detection, immunochromatographic test strip (ICTS)^12^,^13^ presents several distinct advantages, including a user-friendly format, a short period of time to acquire test results, long-term stability over a wide range of climates, and relatively inexpensive production. These characteristics make ICTS an ideal candidate for the on-site primary screening of samples in spot. However, the application of test strip is limited when high sensitivity is needed. Various methods have been reported to improve the sensitivity of test strip, including the design of sensitive equipment to convert signal, the optimization of the test procedure and the improvement of the properties of the probe materials^14^,^15^,^16^. Since both visual inspection signals and quantitative data (optical densities) mainly dependent on the sizes, shapes, components and the surface antibody affinity of probe materials, it is a simple and effective means to improve the detection efficiency and sensitivity by exploring the optimized label probes for ICTS.

In conventional immunochromatographic assay, single-component, solid noble metal particles (e.g., gold nanoparticles) have been used as labeled probes. In the previous works^17^,^18^, it was observed that the bimetallic NPs, especially Ag-based nanostructures could show excellent optical properties to remarkably improve the detection sensitivity in surface-enhanced Raman spectroscopy based sensors. On the other hand, the higher specific surface area of hierarchical or hollow structures can improve the strong surface affinity with antibodies than low-dimensional solid NPs^19^,^20^,^21^,^22^,^23^. Therefore, a bimetallic hollow Ag-Au NPs would have been an ideal choice as labeled probes. To the best of our knowledge, there is no such report using hollow Ag-Au NPs as probes in immunochromatographic test strip. Herein, we synthesized bimetallic, hollow Au-Ag nanoparticles (NPs) via the galvanic replacement reaction, and systematically optimized the size of the Au-Ag NP, hollow size and Au/Ag mole ratio to amplify both the visual inspection signals and the quantitative data (optical densities). Meanwhile, compared with conventional physical interaction between NPs and antibody, we used 11-mercaptoundecanoic acid (MUA) to functionalize the surface of hollow Ag-Au NPs for covalently bonding anti-CLE monoclonal antibody, acquiring highly stable probe materials. The sensitivity of optimized hollow Au-Ag NPs probes for the detection of CLE can be achieved even as low as 2 ppb, which is around 10 fold lower than that of solid Au NP-based test strip.

## Results and Discussion

**Figure 1:**
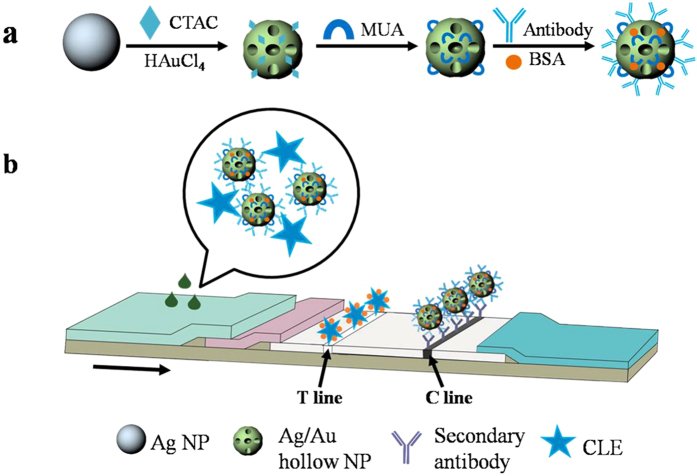
(**a**) The synthesis and surface modification of hollow Au-Ag NPs; (**b**) Schematic illustration of the detection of CLE using hollow Au-Ag NPs-labeled ICTS.

The unique properties of noble metal nanoparticles depend closely on their sizes, shapes and constitutes^24^,^25^. As the crucial probes and signal resource of ICTS, the size, morphology and constitutes of hollow Ag-Au NPs have significant influences on the detection performance of ICTS. Conventionally, solid spherical Au NPs are used commonly as labeled probes of ICTS but the detecting abilities are not the optimal compared with other structured Au NPs^15^. For example, bimetallic, hollow Au-Ag NPs have higher surface area, low cytotoxicity, unique optical properties and well-established bioconjugation strategies^26^,^27^, which make hollow Au-Ag NPs to be a promising candidate for high efficiency labeled probes of TS. In this work, optimized hollow Au-Ag nanoparticles (NPs) were functionalized with specific antibody and used as novel labeled probes for the detection of CLE by ICTS (Fig. 1).

**Figure 2:**
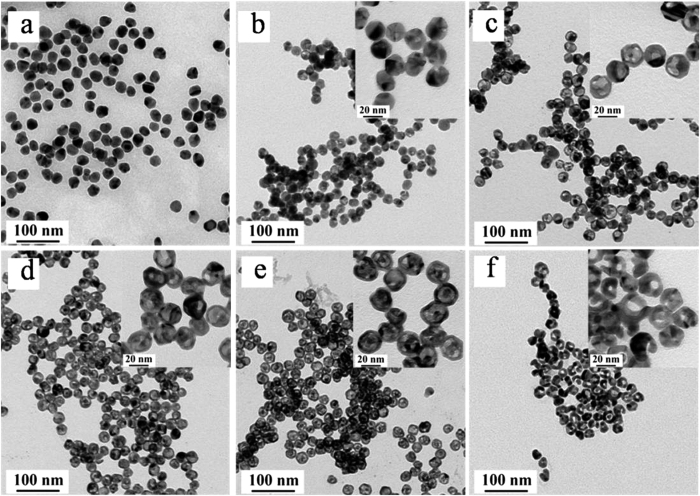
TEM images of Ag NPs (a) and hollow Ag-Au NPs in different growth stages (b–f), the Ag/Au ratios in images (b–f) are about 12.69:1, 4.78:1, 1.77:1, 0.98:1, and 0.46:1, respectively.

Bimetallic hollow Au-Ag NPs are synthesized by galvanic replacement reaction where Ag is replaced by Au since Ag has lower reduction potential^28^. In order to achieve the preferred hollow Au-Ag nanostructures, influence of the ratio of [HAuCl_4_]:[Ag NPs] are systematically studied and successfully synthesized different hollow Au-Ag nanostructures with varying morphologies and compositions. The microstructures of these Au-Ag NPs were visualized by TEM characterization. With the increasing of content of Au, overall sizes of the NPs are not affected much, while the morphology clearly changes from solid to hollow shapes. The sizes of the holes within each NPs were gradually increased with increasing (6 × 10^−5^ to 2.5 × 10^−4^ mmol) content of Au. Careful inspection of the hollow nanocrystals reveals that when Ag NPs (Fig. 2a) react with a small amount of HAuCl_4_ solution, a small hole is observed on the twin boundaries in most of the NPs (Fig. 2b), indicating that the reaction is initiated locally at a high-energy site (i.e. twin boundary) rather than over the entire NP’s surface. As the reaction proceeds, this small-hole serves as the anode, where Ag is oxidized and electrons are stripped. Then the released electrons migrate to the surrounding surfaces and are captured by AuCl_4_^−^, generating Au atoms and deposited on the NP’s surface. As the Au layer forms, the initial small-hole serves as the site for Ag dissolution, facilitating the conversion of the solid NPs into hollow nanostructures (Fig. 2c–e). Much higher amounts (2.5 × 10^−4^ mmol) of HAuCl_4_ solution create larger penetrative holes (~3–5 nm) within the Ag-Au NPs (Fig. 2f). In this way, the surface morphology and aperture sizes of hollow NPs can be controlled by tuning the additive amount of HAuCl_4_ in the reactive solution, which provide the possibility of improving the detecting performance of hollow NPs probes by optimizing the structures of hollow Ag-Au NPs. The morphology of Ag-Au NPs with the Ag/Au ratios of 4.78/1, 0.98/1 and 0.46/1 were further characterized by SEM (Figure S1). The increasing of Au content lead to the larger size of cavity and some Ag-Au NPs finally cracked into incomplete NPs with small size, which is in good agreement with TEM observation.

**Figure 3:**
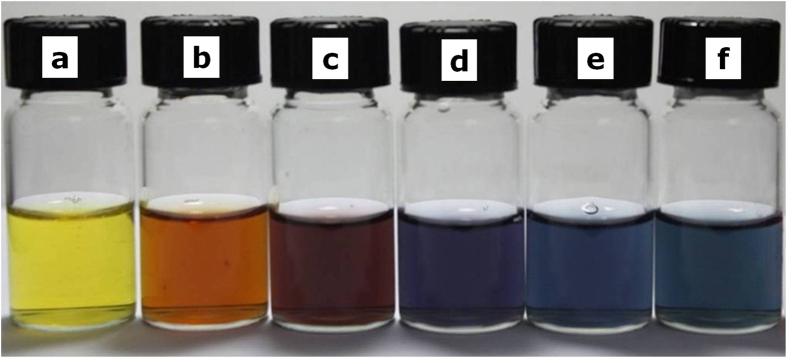
The color changes of Ag-Au porous NPs in the galvanic replacement reaction (a–f) after the addition of different volumes of 0.01 M HAuCl4 from 0, 12, 24, 36, 48 to 50 μL.

**Figure 4:**
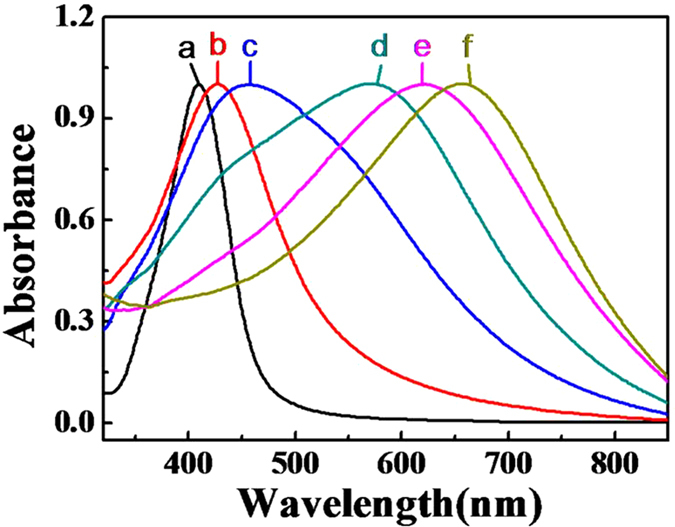
UV/vis absorption spectra of Ag NPs (a) and hollow Ag-Au NPs with different Ag/Au/ratio (b–f): 12.69:1, 4.78:1, 1.77:1, 0.98:1, and 0.46:1.

One of the interesting features of these bimetallic hollow Au-Ag nanostructures is that the color of the NPs strongly depends on the exact amounts of HAuCl_4_ solution. By adding different amounts of the HAuCl4 solution (6 × 10^−5^−2.5 × 10^−4^ mmol) into a fixed volume of Ag NPs, color of the solution varies from yellow to orange, red, purple, blue and finally cyan, as shown in Fig. 3. The optical properties of these hollow Ag-Au NPs solution were studied by UV-vis spectroscopy and shown in Fig. 4. With increasing the amount of HAuCl_4_, the absorption peaks of the solution are noticeably red-shifted from 410 to 657 nm, indicating the surface plasmon resonance shift induced by the increasing of the content Au in the alloy^29^. This red shifting can also be appeared to the different void sizes in the interior, wall thicknesses, and sizes of holes on the walls of the nanostructures. By simply controlling the molar ratio, the surface plasmon peak of the nanostructures can be conveniently tuned over the broad spectral range. The Ag/Au ratios were estimated by EDS (Figure S2) and found that the Ag:Au ratio was gradually reduced from 12.69:1 to 0.46:1 with increasing the additive amount of HAuCl_4_ from 6 × 10^−5^ mmol to 2.5 × 10^−4^ mmol.

These hollow Au-Ag NPs which were stabilized by CTAC were then, functionalized covalently with MUA to endow the NPs with steady negative charged surface (Figure S3) and finally to obtain stable labeled probes^30^. Moreover, negatively charged hollow Ag-Au NPs would be easier to be functionalized with specific antibody for CLE^31^,^32^,^33^,^34^. The as-prepared hollow Ag-Au NPs labeled probes were mixed with different concentrations of CLE samples in an ELISA plate where the probes integrated sufficiently with CLE. Subsequently, the mixed solution was transferred to the TS and migrates through the strip by virtue of capillary force from absorbent pad. The measurement is conducted based on a competitive binding immunoassay^35^. The CLE in the sample competes with the antigen for the limited binding sites of the antibody labeled with marker. When the content of CLE in the sample is below the limit of detection, the Ag-Au hollow NPs-antibody conjugate will be captured by the antigens coated on the test line (T line) on nitrocellulose membrane. A colored T line indicates a negative result^36^. When the content of CLE in the sample is higher than the limit of detection, CLE binds to hollow Ag-Au NPs-antibody conjugate in an ELISA plate, leading to colorless T line in the test region and receiving a positive result (Fig. 2b). Hollow Ag-Au NPs-antibody conjugate were immobilized on control line (C line) coated with goat anti- mouse IgG and form a colored line showing the validity of the ICTS regardless of the presence of CLE.

**Figure 5:**
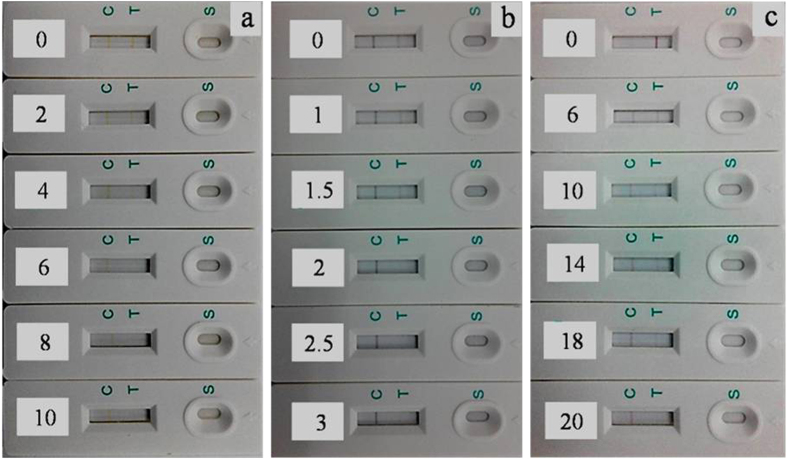
Detection of CLE samples (from 0 to 20 ppb) using Ag NPs (a), Ag-Au porous NPs (b), and Au NPs (c) labeled ICTS.

Different concentrations of CLE analytical standard solution (0−20 ppb) were subjected to the immunochromatographic strip test. Figure 5 shows the visual detection results of CLE samples using hollow Ag-Au NPs probes with Ag/Au ratio of 4.78:1 (Fig. 4c). Besides, common solid Au NPs (Figure S4) and Ag NPs labeled probes with similar sizes are also employed as references to compare their detection performances. The visual inspection limit was defined as the minimum analyte concentration required for showing no apparent color on T line to the naked eye^37^. The detection procedure was repeated three times. Following this definition, the visual qualitative inspection limit of solid Ag NPs probe and Au NPs probes was around 6 ppb and 20 ppb respectively, while the visual detection limit of Ag-Au hollow NPs was approached to 2 ppb (Fig. 5). Compared with solid Au NPs and Ag NPs-labeled test strips, Ag-Au hollow NPs labeled ICTS exhibited much higher sensitivity for qualitatively detecting CLE.

**Figure 6:**
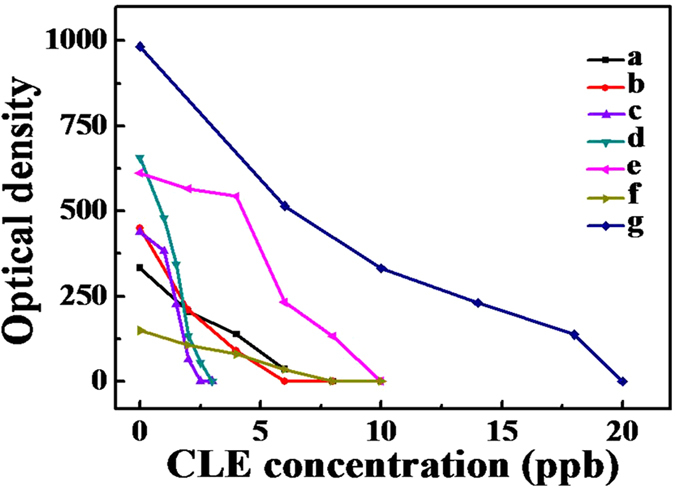
The optical density on the T line in the detection of different concentrations of CLE using Ag NPs and hollow Ag-Au NPs (a–f), and Au NPs (g).

In order to quantitatively study the detection performance of Ag-Au hollow NPs probes, the optical densities of T line were employed to examine precisely the testing properties of ICTS for CLE^37^. Figure 6 shows the optical densities on T line in the detection of different concentrations of CLE using Ag NPs and hollow Ag-Au NPs. Generally, the optical densities decreased gradually with increasing the concentration of CLE. Compared with conventional solid Au NPs, hollow Ag-Au NPs labeled ICTS show higher sensitivity even at lower concentration. Meanwhile, optimal hollow Ag-Au NPs with Ag/Au ratio of 4.78:1 shows strong enough optical density (64 au) on T line even the concentration of CLE was as low as 2 ppb (Fig. 6c), indicating lower quantitative detection limit and more sensitive detection performance than other NPs labeled ICTS^38^. The same detection process was repeated two times, and similar detection limits were obtained, showing good reproducibility.

**Figure 7:**
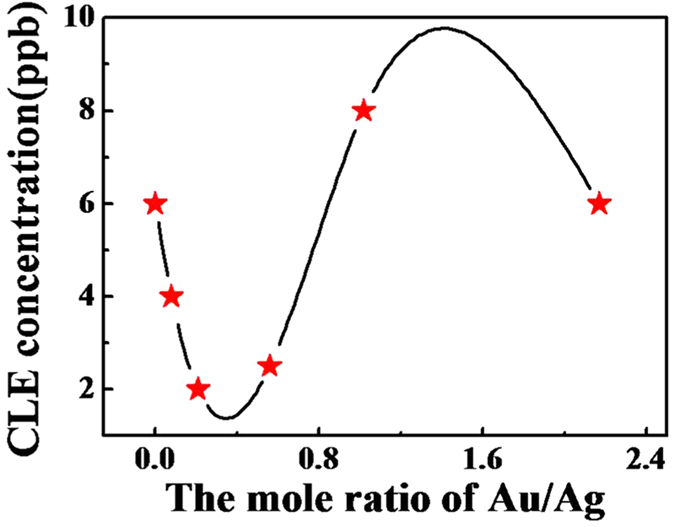
The relationship between the detection limit of CLE and Ag-Au porous NPs with different Ag/Au ratio.

The above results clearly indicated Au/Ag ratio had effects on the detection performance of hollow Ag-Au NPs labeled ICTS. Figure 7 shows the relationship between detection limits and the Ag/Au ratio of hollow Ag-Au NPs. Optimal test strips labeled by hollow Ag-Au NPs with Ag/Au ratio of 4.78:1 displayed the lowest detection limit. It was considered that the Ag/Au ratios and hollow microstructures have influences on the testing behaviors. Compared with solid Au NPs and Ag NPs labeled ICTS, hollow Ag-Au NPs with high surface area and strong conjugation ability can be modified with more antibodies to capture easily target analysts^39^, which improve remarkably the detection properties of ICTS. In addition, Au NPs have localized surface plasmon resonance absorption with longer wavelength than Ag NPs, showing more identifiable optical signal^40^,^41^. The microstructures and Ag/Au ratio have collective influences on the specific detection properties. When the Ag/Au ratio of hollow Ag-Au NPs changes from 12.69:1 to 4.78:1, there are few holes with small size appear on the NPs, having little effect on the surface properties of NPs. It has been observed that the optical properties of Au has dominant influences on the detection results and therefore, hollow Ag-Au NPs (4.78:1) with large ratio of Au exhibits highest sensitivity. On the other hand, oversized apertures such as perforative holes appeared with increasing the Au/Ag ratio and even some Ag-Au NPs broken into small pieces, which destroyed the hollow structures and also reduce the ability of probes capturing analysts. Therefore, hollow Ag-Au NPs with lower Ag/Au ratio and appropriate aperture size exhibit better testing results.

In addition, in order to investigate the selectivity of the prepared clenbuterol-related test strip, other testing analogues or proteins are also detected using hollow Ag-Au NPs labeled probes. β-agonists such as salbutamol, terbutaline, fenoterol, ritodrine, and ractopamine, are most frequently associated with clenbuterol, and their cross-reactivity on clenbuterol strip was examined to test selectivity. Clenbuterol and six kinds of β-agonists at 100 ppb were added on clenbuterol-related test strip respectively. As shown in Fig. S5, the T line on the test strip (No. 1) shows no obvious color while those β-agonists related test strips (No. 2–No. 6) show clear red color on the T line, indicating high specificity of the Clenbuterol-related test strip. Besides, other proteins like bovine serum albumen (BSA), ovum albumin (OVA), casein at a concentration of 10% were added on the clenbuterol-related test strip of No. 7–No. 9, respectively. It is found that red colors were appear obviously on the T line in all the three test strips, showing no detecting performance for referenced proteins. Furthermore, BSA, OVA and casein were mixed respectively with clenbuterol and formed mixing samples with low protein concentration of 2 ppb. No red bands were appeared in the test region (No. 10–No. 12 in Figure S5), displaying good resistance and high selectivity of the strips using hollow Au/Ag NPs as probes.

## Conclusion

In this work, a new type of bimetallic, hollow Ag-Au NPs labeled immunochromatographic test strip has been developed to achieve sensitive and on-site detection of CLE in less than 15 min. The apertures size and Ag/Au ratios are optimized symmetrically by tuning the additive ratio of [HAuCl_4_]:[Ag NPs]. The quantitative detection limit of optimized hollow Ag-Au NPs (Ag/Au ratio of 4.78:1) labeled ICTS approaches to 2 ppb, which is superior over the detection performance of conventional ICTS labeled by solid Au NPs or Ag NPs. Therefore, the optimized hollow Au-Ag NPs labeled test strip can be applied to be an ideal candidate for the rapid screening of CLE in food samples.

## Methods

### Reagents and materials

Chloroauric acid (HAuCl_4_·3H_2_O, 99.9%), tannic acid (C_76_H_52_O_46_), CLE, and goat anti-mouse antibody were purchased from Sigma-Aldrich. Cetyltrimethylammonium chloride (CTAC) was received from Aladdin. Bovine serum albumin (BSA) was purchased from Beijing Xinjingke Biotech Co., Ltd. Sodium citrate Na_3_C_6_H_5_O_7_ and silver nitrate (AgNO_3_) were obtained from Sinopharm Chemical Reagent Co., Ltd. (Shanghai). 11-mercaptoundecanoic acid (MUA) was purchased from J&K Chemical Ltd. (Shanghai). Anti-CLE monoclonal antibody was produced according to our previous work^15^. The sample pad, glass fibre membrane, nitrocellulose membrane, and absorption pad were supplied by Schleicher and Schuell GmbH (Dassel, Germany).

Transmission electron microscopy (TEM) images were acquired on a JEOL JEM 2010 electron microscope operating at 2.0 kV. UV/vis absorption spectra were recorded using a TU-1810 spectrophotometer provided by Purkinje General Instrument Co., Ltd. BioDot XYZ Platform combing motion control with BioJet Quanti3000k dispenser and AirJet Quanti3000k dispenser for solution dispensing were supplied by BioDot (Irvine, CA, USA). SkanFlexi BioAssay strip reader was purchased from Skannex Biotech Co., Ltd. (Oslo, Norway).

### Preparation of Spherical Au NPs

Au NPs with an average diameter of 18 nm were prepared according to our previous reports^42^. Briefly, 100 mL of 2.5 × 10^−4^ M HAuCl4 aqueous solution was heated to 120 °C under vigorous stirring. Then, 10 mL of 1% sodium citrate aqueous solution was injected rapidly into the gold solution. Upon continuous boiling for 20 min, the color of the boiled solution changed to ruby red, indicating the formation of spherical Au NPs. The resultant Au NPs solution was cooled down and stored at 4 °C for further use.

### Preparation of Spherical Ag NPs

Ag NPs were synthesized according to previous report with some modification^43^. Briefly, A 100 mL of aqueous solution containing 5 mM sodium citrate and 0.1 mM tannic acid was heated to boiling under gently stirring. Subsequently, 1 mL of freshly prepared 25 mM AgNO_3_ solution was added into the above solution under vigorous stirring and the color of the mixed solution turned bright yellow. Upon continuous boiling for another 25 min, the resultant Au NPs solution was cooled down and stored at 4 °C for further use.

### Preparation of Au-Ag Hollow NPs

Ag-Au hollow NPs were prepared by the galvanic replacement reaction between Ag NPs and HAuCl_4_ solution^28^. Typically, Ag NPs solution prepared above was centrifuged at 12000 rpm for 20 min and resuspended into equal amount of CTAC solution (80 mM). Then the Au NPs solution was kept at 30 °C without disturbance for 24 h to acquire CTAC-capped Ag NPs solution. Subsequently, 5 mL of CTAC-capped Ag NPs solution was held in a reaction vial and different volumes of HAuCl_4_ solution (12, 24, 36, 48, and 50 μL) was added dropwise into the reaction vial under vigorous stirring. The synthesized Ag-Au hollow NPs products were purified by centrifugation (12000 rpm, 20 min) two times to remove supernatant. The precipitated Ag-Au hollow NPs products were collected and resuspended into equal amount of deionized water.

### Surface Modification of Hollow Au-Ag NPs

A pH-mediated robust method was employed to completely replace CTAC attached on the hollow Au-Ag NPs surface with the MUA molecules^30^. Briefly, 20 mM MUA aqueous solution was prepared by adding dropwise 0.2 M NaOH solution with stirring and periodic sonication to dissolve MUA. Subsequently, 2 mL of freshly prepared MUA solution was added to each 5 mL of purified Ag-Au hollow NPs solutions prepared in section 2.4 with vigorously stirring for 48 h. The MUA-modified Ag-Au hollow NPs products were collected by two rounds of centrifugation at 12000 rpm for 20 min for each round and then redispersed in 2.5 mL of 0.01 M borate buffer at pH 9.

### Surface Functionalization of MUA-modified Ag-Au Hollow NPs with Antibody

The anti-CLE monoclonal antibody solution (200 uL) was added dropwise to 2 mL of the above MUA-modified Ag-Au hollow NPs solution to obtain a final concentration of 2.5 ug ml^−1^ and was stirred for 1 h. Subsequently, 200 uL of 10% (w/v) BSA solution was added to block this solution. After 30 min, the resultant MUA-modified Ag-Au hollow NPs functionalized with antibody was collected by centrifugation and resuspended into 200 uL of borate buffer (pH 9).

### Preparation of Immunochromatographic Test Strips

The sample pad was treated with 50 mM borate buffer (pH 7.4) containing 1% BSA, 0.5% Tween-20, and 0.02% NaN_3_, and then dried at 60 °C for 2 h. CLE–BSA conjugation (0.8 mg ml^−1^) and goat anti-mouse antibody (0.4 mg ml^−1^) were respectively spotted onto the test and control lines on the nitrocellulose membrane, and the dispensed volume was 0.75 mL cm^−1^. The sample pad, glass fiber membrane, nitrocellulose membrane and absorption pad were assembled as a strip.

### Detection of CLE by Immunochromatographic Test Strip Method

CLE samples with different concentrations from 0 to 20 ppb were prepared using sterile 0.01 M PBS (pH 8.5), and mixed with Ag-Au hollow NPs functionalized with antibody complex for 3 min. The resultant mixed solution was transmitted to the test strip and records thetesting results after 10 min using a portable strip reader. All the experiments were performed in triplicate.

β-agonists including salbutamol, terbutaline, fenoterol, ritodrine, and ractopamine or proteins including are added respectively on the CLE-related test strip under the same concentration of 100 ppb and same process. Besides, BSA, OVA and casein were mixed respectively with clenbuterol and formed mixing samples with low protein concentration of 2 ppb.

## Additional Information

**How to cite this article**: Wang, J. *et al*. Hollow Au-Ag Nanoparticles Labeled Immunochromatography Strip for Highly Sensitive Detection of Clenbuterol. *Sci. Rep.* **7**, 41419; doi: 10.1038/srep41419 (2017).

**Publisher's note:** Springer Nature remains neutral with regard to jurisdictional claims in published maps and institutional affiliations.

## Supplementary Material

Supplementary Information
