# Supplementary material for: Hollow Au-Ag Nanoparticles Labeled Immunochromatography Strip for Highly Sensitive Detection of Clenbuterol
Source: Sci Rep. 2017 Jan 30;7:41419. doi: 10.1038/srep41419 (PMC5278391; doi:10.1038/srep41419)
Supplement: Supplementary Information [file srep41419-s1.doc]

**Supporting Information:**

**Hollow Au-Ag Nanoparticles Labeled** **Immunochromatography Strip for Highly Sensitive Detection of Clenbuterol**

*Jingyun Wang, ^1, 2 †^ Lei Zhang, ^2 †^ Youju Huang，[[1]](#footnote-2)*2 Anirban Dandapat,* **^3^***Liwei Dai, ^2^ Ganggang Zhang, ^1,2^ Xuefei Lu,^2^ Jiawei Zhang，^2^ Weihua Lai[[2]](#footnote-3)*1 and Tao Chen[[3]](#footnote-4)*2*

^1^ State Key Laboratory of Food Science and Technology, Nanchang University, Nanchang 330047, China. ^2^ Division of Polymer and Composite Materials, Ningbo Institute of Material Technology and Engineering Chinese Academy of Sciences, No. 1219 Zhongguan West Road, Zhenhai District, Ningbo 315201, China. Fax: (0086)0574-87603570. ^3^Department of Biotechnology, Kumaun University, Bhimtal-263136, Uttarakhand, India


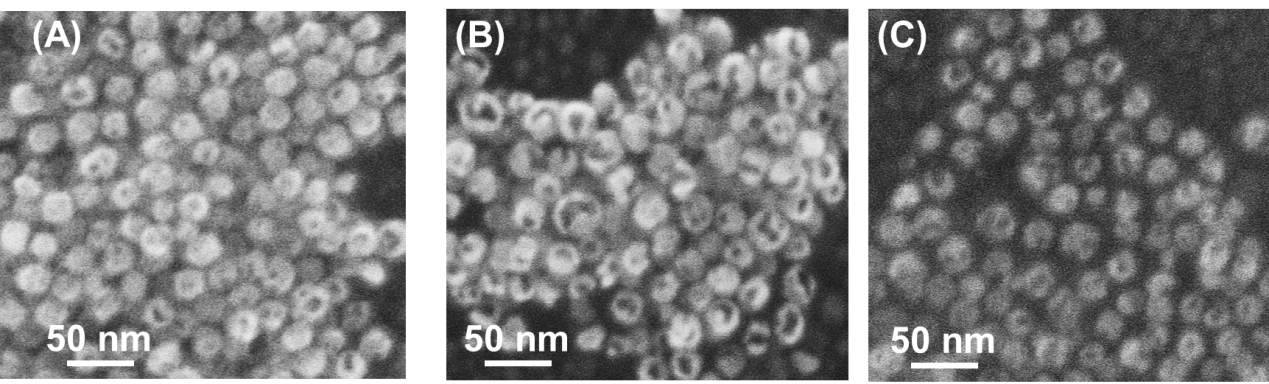


**Figure S1**. The SEM images of hollow Au-Ag NPs with Ag/Au ratios of 4.78/1(A), 0.98/1(B), and 0.46/1(C).


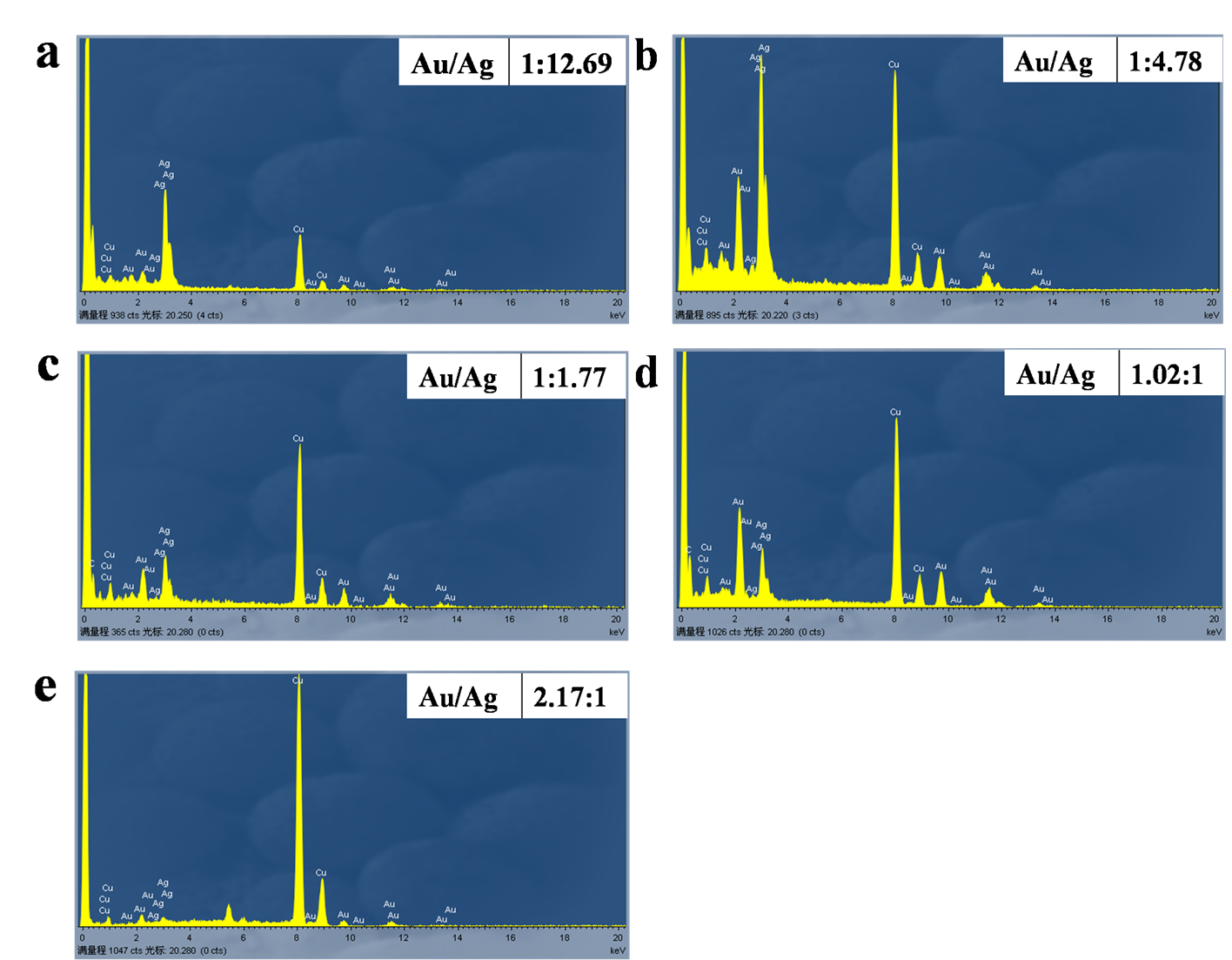


**Figure S2.** EDS analysis for hollow Ag-Au NPs with different Au/Ag mole ratio: 1:12.69, 1:4.78, 1:1.77, 1:0.98, and 1:0.46.

**
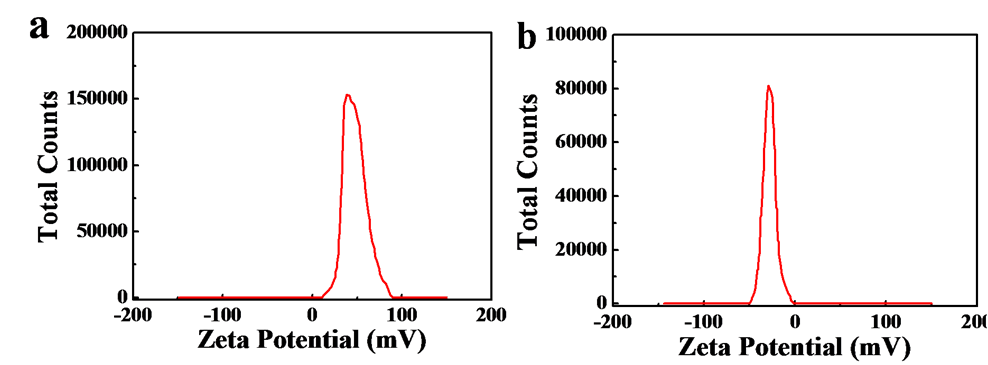
**

**Figure S3.** The ζ- potential of CTAC-capped Ag-Au Porous NPs (a) and MUA-capped Ag-Au Porous NPs.

**
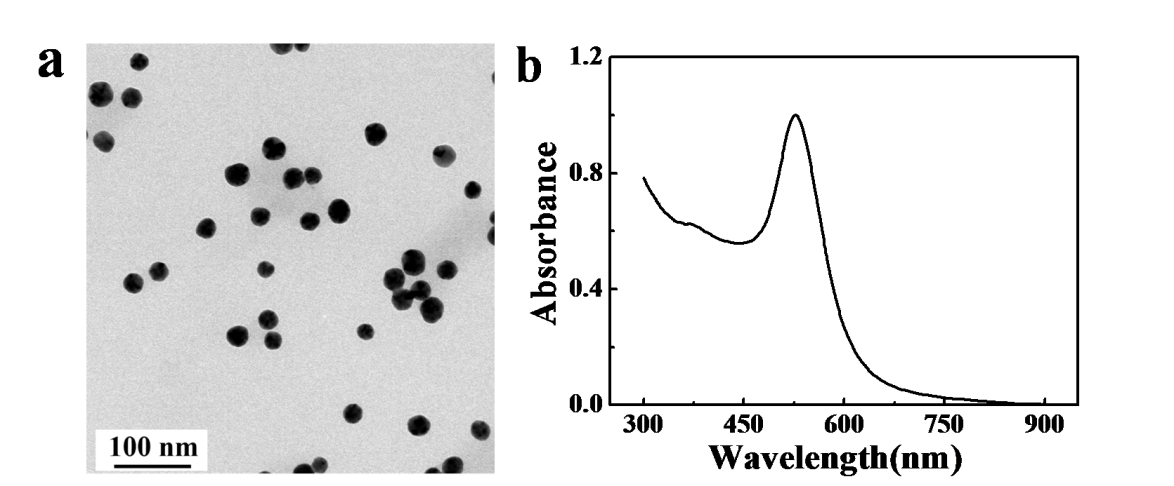
**

**Figure S4.** The characterization of Au NPs. TEM images of Au NPs (a), and UV-visible absorbance spectra of Au NPs (b).


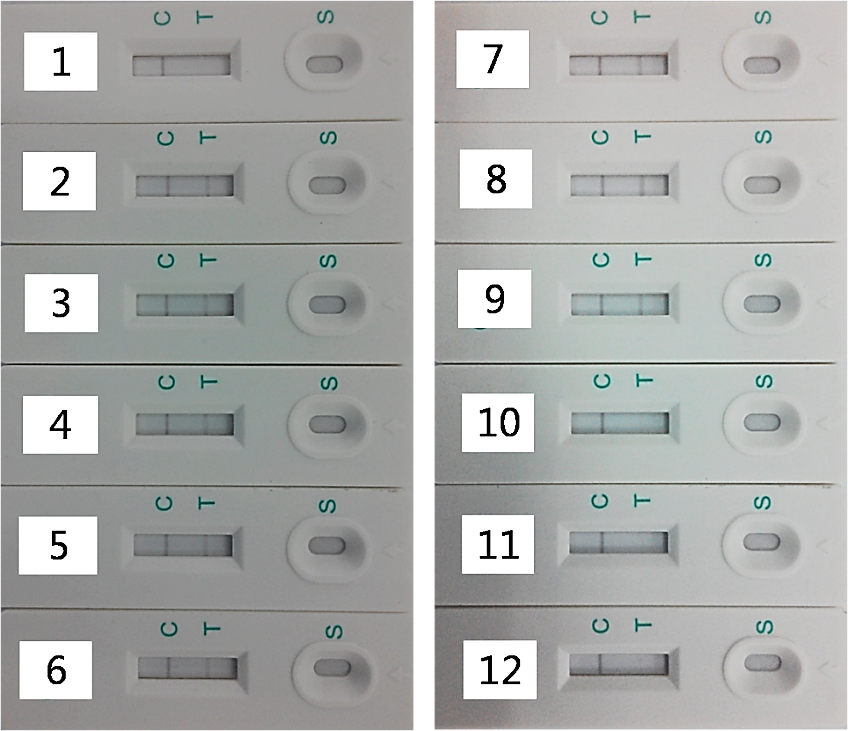


**Figure S5.** Specificity of test strip. No.1-No.6 represent Clenbuterol, Salbutamol, Terbutaline, Fenoterol, Ritodrine, and Ractopamine added test stripes, respectively.; No.7-No.9 represent BSA, OVA and Casein added test stripes, respectively.; No.10- No.12 represent BSA+Clenbuterol, OVA+Clenbuterol, Casein+ Clenbuterol added test stripes, respectively.

1. ^^ [↑](#footnote-ref-2)
2. ^^Corresponding Authors: E-mail: [yjhuang@nimte.ac.cn](mailto:yjhuang@nimte.ac.cn); [talktolaiwh@163.com](mailto:talktolaiwh@163.com) and [tao.chen@nimte.ac.cn](mailto:tao.chen@nimte.ac.cn). [↑](#footnote-ref-3)
3. ^^ [↑](#footnote-ref-4)
